# Dive characteristics can predict foraging success in Australian fur seals (*Arctocephalus pusillus doriferus*) as validated by animal-borne video

**DOI:** 10.1242/bio.016659

**Published:** 2016-02-12

**Authors:** Beth L. Volpov, David A. S. Rosen, Andrew J. Hoskins, Holly J. Lourie, Nicole Dorville, Alastair M. M. Baylis, Kathryn E. Wheatley, Greg Marshall, Kyler Abernathy, Jayson Semmens, Mark A. Hindell, John P. Y. Arnould

**Affiliations:** 1 School of Life and Environmental Sciences, Deakin UniversityBurwood, Victoria 3125, Australia; 2 Marine Mammal Research Unit, Institute for the Oceans and Fisheries, University of British Columbia, Vancouver, British Columbia V6T 1Z4, Canada; 3 Remote Imaging Department, National Geographic, Washington, DC 20036, USA; 4 Institute for Marine and Antarctic Studies, University of Tasmania, Hobart, Tasmania 7001, Australia

**Keywords:** Crittercam, Foraging behaviour, Animal-borne video, Dive profile analysis

## Abstract

Dive characteristics and dive shape are often used to infer foraging success in pinnipeds. However, these inferences have not been directly validated in the field with video, and it remains unclear if this method can be applied to benthic foraging animals. This study assessed the ability of dive characteristics from time-depth recorders (TDR) to predict attempted prey capture events (APC) that were directly observed on animal-borne video in Australian fur seals (*Arctocephalus pusillus doriferus*, *n*=11). The most parsimonious model predicting the probability of a dive with ≥1 APC on video included only descent rate as a predictor variable. The majority (94%) of the 389 total APC were successful, and the majority of the dives (68%) contained at least one successful APC. The best model predicting these successful dives included descent rate as a predictor. Comparisons of the TDR model predictions to video yielded a maximum accuracy of 77.5% in classifying dives as either APC or non-APC or 77.1% in classifying dives as successful verses unsuccessful. Foraging intensity, measured as either total APC per dive or total successful APC per dive, was best predicted by bottom duration and ascent rate. The accuracy in predicting total APC per dive varied based on the number of APC per dive with maximum accuracy occurring at 1 APC for both total (54%) and only successful APC (52%). Results from this study linking verified foraging dives to dive characteristics potentially opens the door to decades of historical TDR datasets across several otariid species.

## Abstract (precis)

**Summary:** We assessed the ability of dive characteristics in Australian fur seals to predict foraging behavior and success that were directly observed on video. TDR data was able to reliably predict presence of prey as well as total successful prey captured per dive.

## INTRODUCTION

Successful foraging is one of the most basic determinants of individual survival and drives the dynamics of populations (e.g. Pistorius et al., 2004). Accordingly, ecologists have long been interested in quantifying foraging success (Austin et al., 2006a,b; Dragon et al., 2012; Lesage et al., 1999; Robinson et al., 2010). However, for marine predators, determining foraging success is inherently difficult due to the limited ability to directly observe them underwater. Consequently, researchers rely upon biologging tags attached to the animals to collect information from which prey encounters or capture events can be inferred.

One of the most commonly used biologging devices on marine mammals is the time-depth recorder (TDR), which can provide relatively inexpensive, easily interpretable data. In addition, given that TDRs have been used to describe characteristics of the diving behavior of marine mammals for over 50 years (Kooyman, 1965), there is a wealth of ‘historical’ TDR data that is available on a wide range of pinniped species for re-analysis using new methodologies. Data from TDRs have been employed as indirect measures of foraging behavior, primarily focusing on 2D dive profiles or ‘dive shapes’ in a wide range of pinnipeds and seabirds (Austin et al., 2006b; Bengtson and Stewart, 1992; Hindell et al., 1991; Le Boeuf et al., 1988; Lesage et al., 1999; Schreer et al., 2001; Schreer and Testa, 1996). Although dive shape analysis has been widely implemented in the last ∼30 years, dive classification protocols vary widely ranging from subjective manual analysis to rigorous statistical analysis (for a review see Schreer et al., 2001).

In the absence of direct evidence of foraging success, dives with longer bottom phase durations that are U-shaped are often inferred to indicate foraging success (e.g. Gallon et al., 2013). Inferences from dive shape analysis are supported by correlations with stomach sensor pills (which are inferences themselves) on several species of pinnipeds (Horsburgh et al., 2008; Kuhn and Costa, 2006; Lesage et al., 1999), but few studies have directly linked dive characteristics to confirmed foraging success in free-ranging animals (e.g. Madden et al., 2008). Without direct validation, it remains unclear if dive shape or other dive characteristics can be used to differentiate successful foraging from unsuccessful foraging in diving pinnipeds.

For benthic foraging species, such as the Australian fur seal (*Arctocephalus pusillus doriferus)*, 2D dive shape is not an appropriate metric to predict successful foraging due to the lack of dive shape variability. Unique among fur seals (although similar to sea lions), Australian fur seals are predominately benthic foragers with the majority (78-85%) of dives classified as U-shaped and at maximum depth (<100 m) corresponding to bathymetry of Bass Strait (Arnould and Hindell, 2001; Arnould and Kirkwood, 2007; Hoskins et al., 2015). While temporal and spatial changes in foraging intensity have been observed in this species and used to infer important foraging zones (Hoskins et al., 2015), it is still not known whether these regions correspond to areas of foraging success. Additional information, therefore, is needed to use dive characteristics in predicting foraging success and distinguishing successful foraging attempts in Australian fur seals and other benthic foragers, which may lack diversity in dive shape.

Animal-borne imaging devices (still or video cameras) have been used for direct observation of foraging success in free-ranging pinnipeds, seabirds, and marine turtles (Bowen et al., 2002; Davis et al., 1999; Hooker et al., 2002, 2008; Iwata et al., 2012; Naito et al., 2010; Parrish et al., 2005; Thomson et al., 2011; Watanabe and Takahashi, 2013). In the absence of direct evidence of foraging from video, stomach sensor pills or accelerometers have been used to infer foraging success. For example, drops in stomach temperature have been used to estimate prey ingestion in pinnipeds (Andrews, 1998), but this method is limited by the large size of the pill animals must ingest, short retention times (Horsburgh et al., 2008; Lesage et al., 1999), and inconclusive accuracy estimates (Bekkby and Bjørge, 1998; Boyd et al., 2010; Hedd et al., 1996; Kuhn and Costa, 2006).

Back-mounted accelerometers have been used to provide general measures of activity (Wilson et al., 2006) or reconstruct fine-scale underwater movements (e.g. Shepard et al., 2008) that may indicate general foraging activity but not necessarily specific prey captures. Head-mounted accelerometers have been used to estimate attempted prey captures (APC) in diving vertebrates (Carroll et al., 2014; Skinner et al., 2009; Suzuki et al., 2009; Viviant et al., 2014, 2010; Volpov et al., 2015; Watanabe and Takahashi, 2013; Ydesen et al., 2014). However, only two of these studies directly validated this method on free-ranging animals consuming multiple prey types (Volpov et al., 2015; Watanabe and Takahashi, 2013), and the ability of this method to accurately distinguish between successful prey captures or missed attempts remains unclear.

This study examined the relationships between dive characteristics and known APC (determined via animal-borne video) in Australian fur seals to determine if TDR data alone can reliably predict foraging behavior or foraging success. Specifically, this study determined (1) if dive characteristics can reliably predict the probability of prey presence in APC versus non-APC dives, (2) if dive characteristics can reliably predict the probability of a dive with at least one successful APC present, (3) whether dive characteristics can predict total APC per dive, and (4) if dive characteristics could predict total successful APC per dive. This was the first study to verify inferences of foraging from TDRs with simultaneous animal-borne video in a benthic forager that lacks variability in dive shape.

## RESULTS

### Summary of dive characteristics

The ranges and distributions of dive characteristics for APC and non-APC dives were similar (Table 1). The majority of dives occurred at night (77%). Within all of the training dives, 68.4% of the dives had at least one successful APC, 30.4% were unsuccessful because it was a non-APC dive, and 3.8% were unsuccessful because they only contained missed capture attempts. All dives analyzed were classified as U-shaped without wiggles present using the scheme of Arnould and Hindell (2001) and were divided into the phases of pre-dive surface interval, descent, bottom, ascent, and post-dive surface interval to permit calculation of the selected focus variables.

**Table 1.**
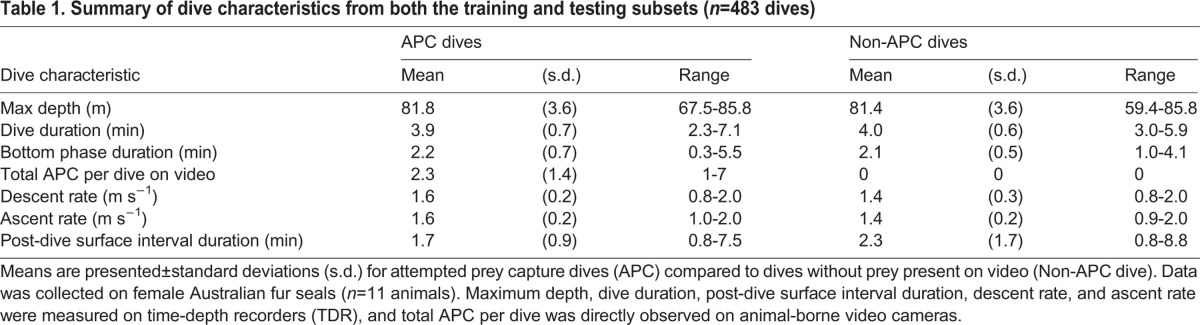
**Summary of dive characteristics from both the training and testing subsets (*n*=483 dives)**

Within the APC dives, 74% of the dives exclusively targeted fish (i.e. seals attempted to capture only fish on entire dive). The remaining 26% of the APC dives had mixed prey items, including various combinations of fish, cephalopods, stingrays, small sharks, and unknown prey. All dives that had one or more octopuses (2.6%), squids (0.6%), stingrays (0.8%), sharks (1.4%), or unknown prey (20.6%) also had at least one fish observed on video. Due to the low diversity in prey type, inclusion of prey type either as a predictor variable in the GLMMs and GAMMs was not possible.

### Comparison of dive characteristics between APC vs non-APC dives

As previously mentioned, models could not be tested that contained both dive duration and bottom duration due to co-linearity. The GLMM results for predicting APC or non-APC dive type were similar when the full model was constructed with either dive duration or bottom duration on the training subset (Table 2). Subsequent data analysis for predicting dive type focused on dive duration because it is more easily obtained from raw TDR records. Models including maximum dive depth would not converge due to large eigenvalue ratios and, therefore, maximum dive depth was excluded from further analysis. This result was likely due to the low variability in maximum depth (Table 1) attributable to the predominately benthic foraging behavior and the low variation in seafloor depth in Bass Strait (Arnould and Hindell, 2001; Arnould and Kirkwood, 2007; Hoskins et al., 2015). The maximum dive depths observed in our study corresponded to maximum depths reported for Bass Strait (Murray and Parslow, 1999), and video data confirmed that the seals dived to the seafloor on all dives analyzed.

**Table 2.**
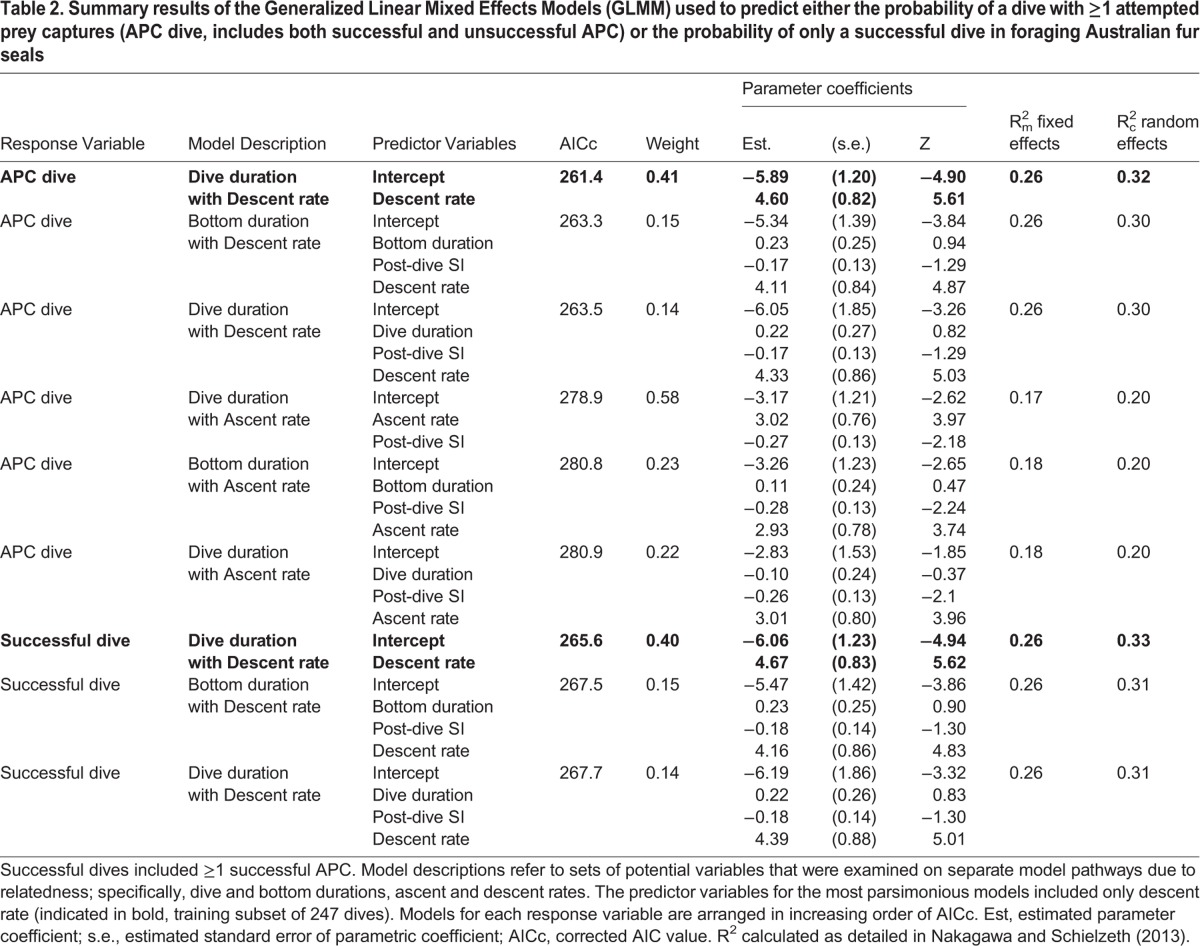
**Summary results of the Generalized Linear Mixed Effects Models (GLMM) used to predict either the probability of a dive with ≥1 attempted prey captures (APC dive, includes both successful and unsuccessful APC) or the probability of only a successful dive in foraging Australian fur seals**

**Fig. 1.**
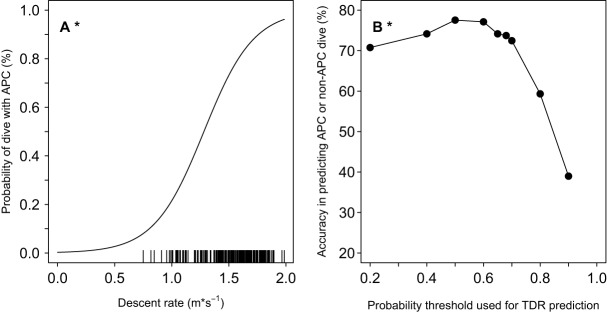
**Probability of a dive with ≥1 attempted prey captures (APC) in response to descent rate and accuracy of the GLMM relative to animal-borne video.** (A) The most parsimonious model on the training subset included descent rate as predictive variable (Table 2). Distribution of descent rate is indicated with a rug plot. (B) Accuracy was calculated as the percent of dives correctly predicted as either APC or non-APC on the testing subset of dives (Table S1).

The most parsimonious model predicting the probability of an APC dive included only descent rate as a predictor variable (Table 2, Fig. 1A). Neither dive duration nor post-dive surface interval significantly improved the model's ability to reliably predict whether the dive included an APC (Table 2). Ascent rate improved the full model, but descent rate remained the best predictor variable on the training subset as indicated by lower AICc and greater R^2^_m_ and R^2^_c_ values (Table 2).

In order to assess accuracy of using TDR data to predict foraging behavior on dives without video available, the most parsimonious model with descent rate generated from the training subset was used to predict the probability of an APC dive on the testing subset, and then compared it to known APC dive classification determined via video (Fig. 1B). Accuracy was defined as the percentage of dives correctly predicted by the model as APC or non-APC dives relative to video classification (Eqn 1, range=39.0 to 77.5%). As the probability threshold used to predict dive type from TDR increased, the accuracy increased slightly, hit an inflection point at 0.50, and then decreased sharply. The inflection point at the 0.50 probability threshold represented the maximum accuracy in predicting APC dives from descent rate (77.5%, Fig. 1B; Table S1).

### Comparison of dive characteristics between successful vs unsuccessful dives

**Fig. 2.**
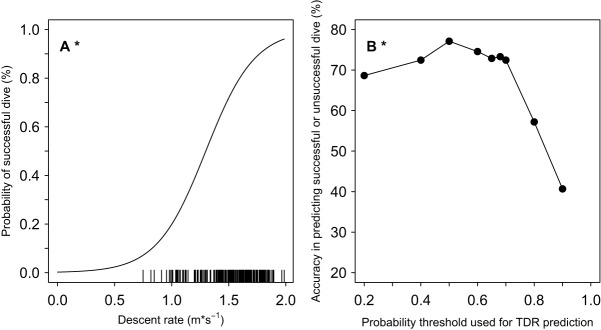
**Probability of a successful dive in response to dive characteristics and accuracy of the GLMM relative to animal-borne video.** (A) The most parsimonious model included descent rate as a predictor variable on the training subset (Table 2). Successful dives had at least one successful attempted prey capture (APC) per dive. Distributions are indicated with a rug plots. (B) Accuracy was calculated as the percent of dives correctly predicted as either successful or unsuccessful on the testing subset of dives (Table S2).

The GLMM results for predicting successful or unsuccessful dive type had lower AICc values when the full model included decent rate (all AICc<268) compared to ascent rate (all AICc>284, Table 2). Consequently, only the models with descent rate are presented for successful dives (Table 2). The most parsimonious model predicting the probability of a successful dive from within all of the dives (APC and non-APC combined) included descent rate (Table 2, Fig. 2A). As descent rate increased, the probability of a successful dive increased (Fig. 2A). Similar to the GLMM on APC dives, AICc values were similar when the full model was constructed with either dive duration or bottom duration in predicting successful dives (Table 2). Accuracy in predicting successful or unsuccessful dives ranged from 40.7 to 77.1% (Table S2; Fig. 2B), and accuracy trends were nearly identical to those in predicting APC dives (Fig. 1B). Maximum accuracy for predicting successful or unsuccessful dives occurred at the 0.50 probability threshold (77.5%, Fig. 2B; Table S2).

### Analysis of total APC per dive as an estimate of foraging intensity

**Fig. 3.**
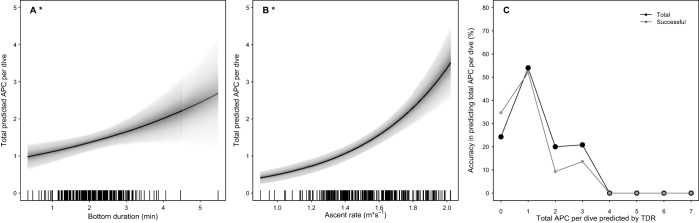
**Total expected attempted prey captures (APC) per dive in response to bottom duration and ascent rate and accuracy of the GAMM relative to animal-borne video.** (A,B) The most parsimonious model included bottom duration (A) and ascent rate (B) as predictors on the training subset of dives (Table 3). Distributions of bottom duration and ascent rate are indicated with a rug plots, and grey bands represent 95% confidence intervals around the predicted response. (C) Accuracy was calculated as the percent of either total APC or only successful APC predicted correctly out of the total APC on the testing subset (Table S3).

Mean total APC per dive was 2.3±1.4 for the APC dives and 1.5±1.5 with APC and non-APC dives combined (Table 1). Models predicting total APC per dive that included maximum dive depth all had greater AICc values than the counterparts without depth included; therefore maximum dive depth was not included in the presented results. GAMM results showed that the most parsimonious model predicting the total APC per dive included bottom duration and ascent rate as significant predictor variables (Table 3, Fig. 3A,B, *n*=247 dives). As bottom phase duration and ascent rate increased, the expected APC per dive increased (Fig. 3A,B). The greatest number of APC per dive occurred on dives with approximately >4 min duration and >1.8 m s^−1^ ascent rate. The model including smoothed descent rate and linear bottom duration was also useful in predicting total APC per dive, but had a higher AICc than the corresponding model with linear ascent rate and bottom duration (656.8 vs 649.8, Table 3). The full model including smoothed terms of bottom duration, post-dive surface interval, and ascent rate (AICc=655.9) was slightly lower than the corresponding model including dive duration (AICc=656.9, Table 3). This indicated that bottom duration was a better predictor of total APC per dive than dive duration. Neither post-dive surface interval, descent rate, nor dive duration improved the model's ability to predict total APC per dive compared to the final model with bottom duration and ascent rate (both not smoothed, Table 3).

**Table 3.**
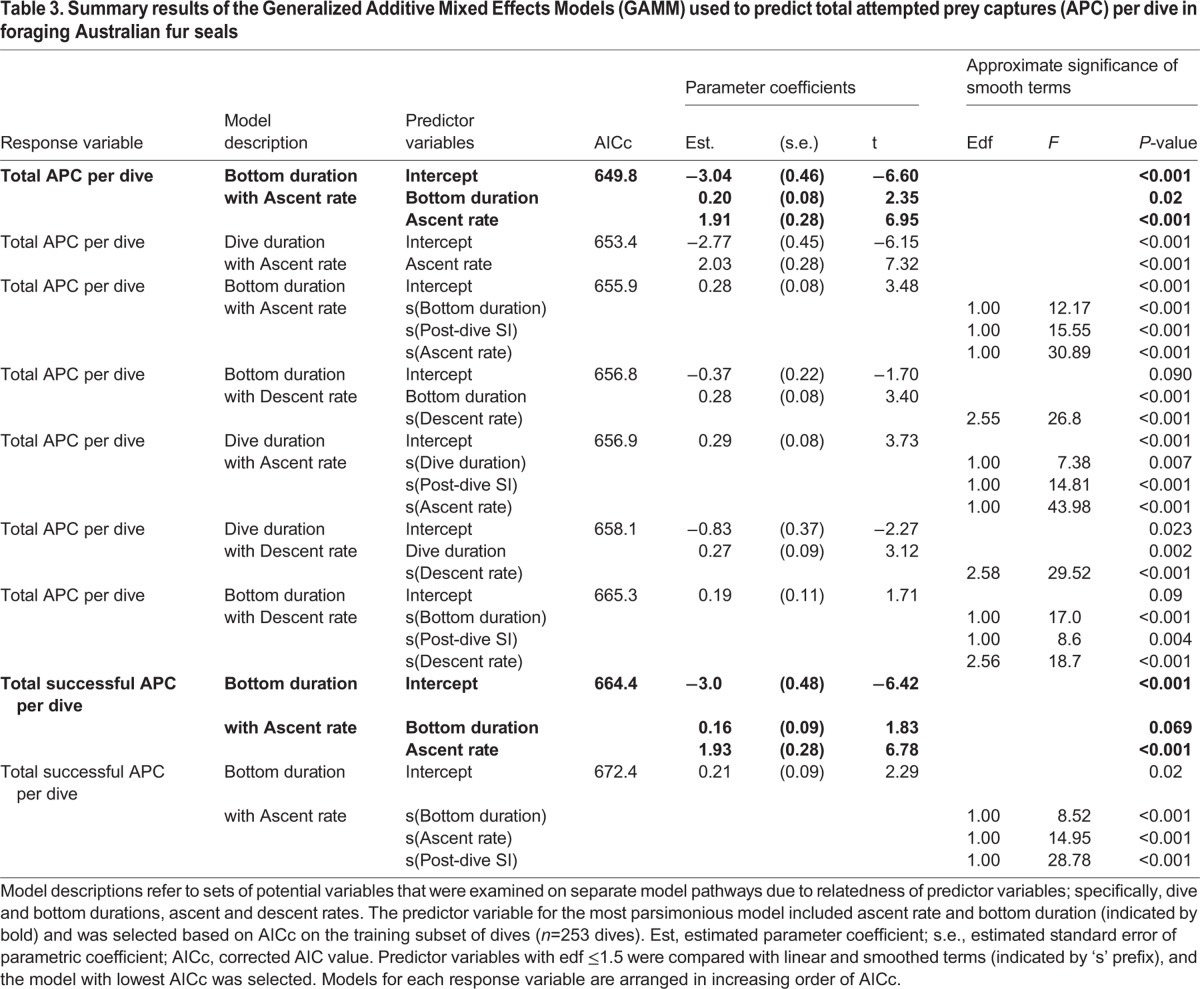
**Summary results of the Generalized Additive Mixed Effects Models (GAMM) used to predict total attempted prey captures (APC) per dive in foraging Australian fur seals**

Results showed that the majority of the individual APC events were successful (93.5% of 389 total APC in the training subset). GAMM results showed that the most parsimonious model predicting total successful APC per dive included ascent rate and bottom duration as non-smoothed terms (AICc=664.4, data included all training dives with 0-7 APC per dive, Table 3, Fig. 3A,B). The model with ascent rate and bottom duration as smoothed terms did not improve the ability to predict total successful APC (AICc=668.7). The accuracy in predicting the total number of APC per dive with bottom duration and ascent rate varied depending on the number of predicted APC per dive (Fig. 3C; Table S3). The accuracy values and trends in predicting the total successful APC per dive were slightly greater than that of predicting total APC (Fig. 3C). The maximum accuracy was observed within dives that had only 1 APC on video for both the total APC (54.1%) and only the successful APC (52.0%, Fig. 3C). The accuracy across all categories (0-7 APC) was similar for total successful APC (13.7±19.6%) compared for total APC (14.9±19.2). Mean values across multiple categories were impacted by the 0% accuracy for the less common 4-7 APC. The salient point is that for the most common number of APC per dive across all dives (1 APC per dive), the GAMM models had 54.1% accuracy in predicting total APC per dive and 52.0% in predicting only the successful APC (Table S3; Fig. 3C).

## DISCUSSION

Foraging success is a key measure of individual bioenergetics and influences reproductive success and population growth. This study used video validation to assess the ability of TDR data alone to reliably predict foraging behavior and success. This included the ability of TDR data to determine whether individual dives contained APCs, whether individual foraging dives were successful, and the total number of successful APC.

### Comparisons among APC, non-APC, and successful dives

Neither dive duration nor bottom duration improved the prediction of APC dives (Table 2). Models of optimal dive theory predict that a seal should terminate a dive earlier when there are no prey present (i.e. shorter dive durations for absence of prey; Thompson and Fedak, 2001). This suggests that dives with greater prey density should be longer in duration than dives without prey present. However, our results demonstrated that dive duration was not a significant predictor of APC dives. This is likely because Australian fur seals are benthic foragers and optimal foraging models indicate the benefit of terminating a dive early is reduced for deeper dives and benthic foragers (Thompson and Fedak, 2001). For example, elephant seals (*Mirounga leonina*) have been reported to have similar dive durations when prey was present or absent (Gallon et al., 2013), likely because all of their foraging are associated with long, deep dives. However, in Weddell seals (*Leptonychotes weddellii*), dive duration, depth, and speed are all significant predictors of foraging success (Davis et al., 2003). Therefore, the low predictive power of dive duration in the current study was likely influenced by the moderate probability of success (68.4% of all training dives had at least one successful APC present) as noted in Thompson and Fedak (2001). Optimal foraging theory also suggests that bottom duration should change with depth. However, there was almost no variation in maximum depth and, not surprisingly, this was not useful in distinguishing between APC and non-APC dives.

Descent rate alone was the best predictor of a dive that involved an APC (Fig. 1, Table 2) and also of successful dives where at least one prey was consumed. Our results from the GLMMs suggested seals were modifying descent rate based on real-time evaluation of the success of the previous dives. This implies that seals were anticipating success on the next dive based on a coarse presence or absence evaluation of the previous dives and, thus, increasing descent rate to get back down to the profitable prey patch. This hypothesis is supported by recent spatial-temporal analysis of Australian fur seal diving behavior in Bass Strait (Hoskins and Arnould, 2013; Hoskins et al., 2015). Using first passage time analysis that predicts areas of foraging intensity, Hoskins et al. (2015) reported faster ascent and descent rates with reduced post-dive surface intervals for dives with increased foraging intensity. Our results concurred, with increased transit rates for models predicting both prey presence and successful dives, but post-dive surface interval duration was not an important predictor in the current study. Additionally, animals could have been modifying other dive characteristics such as angle of descent rather than descent rate as observed in penguins (Ropert-Coudert et al., 2001; Ropert-Coudert et al., 2006).

### Analysis of total APC per dive as an estimate of foraging intensity

While descent rate was the primary predictor of prey presence in a dive, the most useful predictors of foraging success were different within a dive after the seals reached the foraging zone. GAMM results suggested that the seals in the current study were modifying bottom duration and ascent rate based on assessments of the current success of a given dive (i.e. real-time evaluation). Within a dive, the seals continued to alter behavior to maximize success (total APC consumed) based on prey encounter rate or possibly a total prey per dive ‘cut-off’. Optimal diving theory predicts that total prey encountered will increase linearly as time in the foraging zone increases (Hooker et al., 2002; Kramer, 1988). Indeed, as predicted seals in this study made more total APCs on dives with longer bottom durations (Fig. 3A). Real-time evaluation and changes in dive behavior in response to increased prey density has also been demonstrated in trained Steller sea lions (Goundie et al., 2015) and in wild Antarctic fur seals in response to inferred foraging rate (Iwata et al., 2015).

Dives with faster ascent rates had more predicted total APC per dive (Fig. 3B). Transit rates (descent and ascent) were also found to be the best predictor of APC in free-ranging Antarctic fur seals at multiple time-scales ranging from a single dive, over several hours, or over a complete night (Viviant et al., 2014). It should be noted, that the APCs of the Antarctic fur seals (*Arctocephalus gazella*) were estimated from a technique calibrated on trained Steller sea lions. For our study, it is not likely that larger prey brought to the surface by the seals substantially influenced the ability of ascent rate to reliably predict total APC per dive. While 37% of the dives within the training subset had at least one prey item consumed on ascent or at the surface, mean ascent rates for these dives were similar (1.60±0.24) to dives when all prey was consumed during the bottom phase (1.50±0.22, LRT=2.80, *P*=0.09). Although rare, all cephalopods were consumed at the surface, and their consumption immediately ended a dive in all observed instances. The current study lacked the prey diversity to fully assess the potential influence of prey type on ascent rate and total APC per dive. Future research could explore this by analyzing a wider range of prey types across multiple seasons.

The majority (94%) of APC in the training subset were successful; therefore, the analysis of successful APC followed similar trends to that of total APC (*n*=389 total APC). As bottom duration increased, the total successful APC also increased. This is in contrast to other studies which have predicted that dives with successful APC would be shorter in duration based on increased energetic requirements to capture, handle, and potentially digest prey (Williams et al., 2004). These predictions partially assume that animals digest while foraging but the extent that digestion occurs while otariids are diving remains unclear (Rosen et al., 2015; Sparling et al., 2007).

### Field applications

This study compared the predicted dive type or total APC from the models to the actual values on video to assess the model accuracy on the testing subset of dives (Fig. 1B, Fig. 2C, Fig. 3C; Tables S1,S2,S3). Our goal was to validate a classification method that could be easily applied to future and historical TDR datasets to assess the accuracy of using TDR data to reliably predict foraging success in Australian fur seals. Overall the ‘best’ models were achieved using descent rate for predicting APC dives or bottom duration and ascent rate to predict total and successful APC. Descent rate can reliably predict APC dives reasonably well depending on the probability threshold selected with a maximum accuracy of 77.5% at 0.50 probability threshold (Fig. 1B; Table S1).

To illustrate how this technique can be applied to novel data and what new information might be obtained from larger datasets with only TDR data, the GLMMs and GAMMs were applied to a larger database of dives collected on the same animals. Given that 68% of the total test subset dives were successful, and that both GLMMs had the same maximum accuracy at 0.50 probabilities, the model with APC dive type was used to predict onto the larger TDR dataset. The GLMM used descent rate as a predictor variable (as determined from dives with video and TDR) to estimate how many of the total dives were APC dives within the total dives with TDR data (i.e. not limited by video, *n*=3352 dives with TDR). This larger database also included the previous 483 dives from the training and testing subset. The proportion of total dives that were predicted as an APC dive using descent rate varied among animals and was influenced by the total dives per animal (24.8-100%, mean=77.5±23.3%, Table 4). Across all animals combined, 79.6% of the 3352 total dives were predicted as APC dives.

**Table 4.**
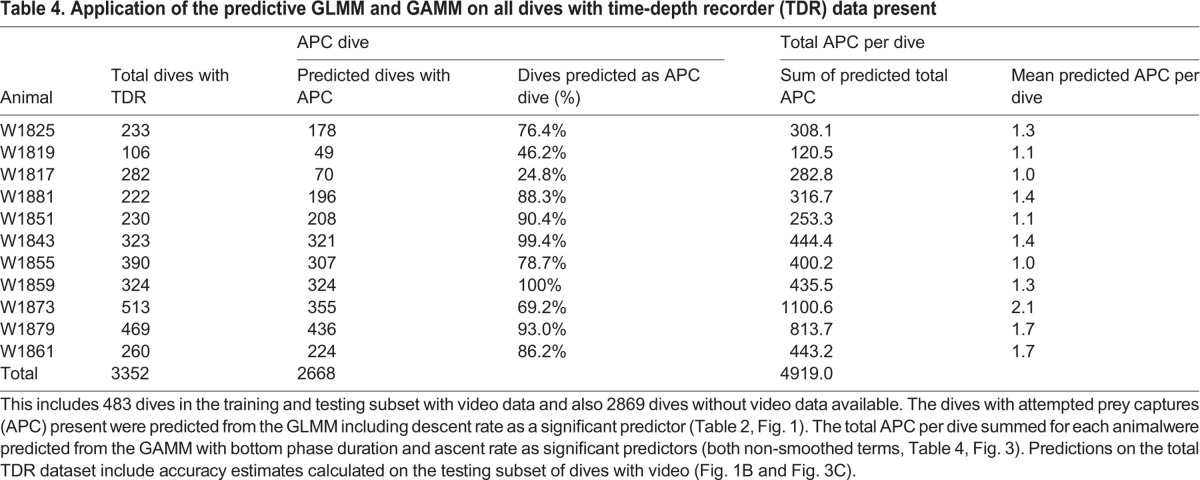
**Application of the predictive GLMM and GAMM on all dives with time-depth recorder (TDR) data present**

The maximum accuracy in predicting the total number of APC per dive from the GAMM (54.1%) or total successful APC per dive (52.0%, Fig. 3C; Table S3) was less than the maximum accuracy for predicting APC dives from the GLMM (77.5%, Fig. 1B). It is reasonable that the more detailed the response variable being predicted, the greater the error in that prediction because there are more potential or more complicated outcomes. Therefore it is logical that the error in predicting APC dives (less detail with only two categories) is greater than the error in predicting more detailed total APC per dive (up to seven categories).

Given that 68.4% of the total APC were successful within the training dives, and that the total APC model had 2.1% greater accuracy compared to only the successful APC, the total APC model was used to predict the larger TDR dataset instead of the successful APC model. The GAMM that included bottom duration and ascent rate predicted a total of 4919 APC in 3352 dives across all animals (Table 4). We adjusted the sum of the predicted total APC by the total dives with TDR per animal to yield a ‘mean predicted APC per dive’ per animal. The mean predicted APC per dive ranged from 1.0 to 2.2 and corresponded to the mean APC per dive directly observed on video (2.3, Table 1). Caution should be used when interpreting the predictions on all the larger TDR datasets because the model carries a wide range of accuracy estimates depending on the total number of APC per dive (Fig. 3C; Table S3).

### Conclusion

The present study used concurrent video and TDR data to identify characteristics in the TDR data that could reliably predict the presence or absence of APC, successful dives, total APC, and total successful APC with quantified accuracy estimates. However, similar dive shapes did not indicate similar dive function or behaviors as all of the non-APC dives with prey absent were U-shaped. Results demonstrated that most useful predictor variables differed depending on the data resolution of the response variable (APC dive type verses total APC per dive), as also indicated in previous research (Austin et al., 2006b; Viviant et al., 2014). Despite the lack of variation in dive shape and maximum dive depth, TDRs were able to distinguish between APC and non-APC dives using descent rate with a reasonable accuracy of up to 77.5%. Dive characteristics were also able to identify dives that had at least one successful APC with accuracy up to 77.1%. Seals had greater foraging intensity and captured more total prey on dives with longer bottom duration and faster ascent rates. However, the accuracy in predicting the total APC per dive (0-52%) was variable depending on the number of APC per dive (0-7). Future research including greater prey diversity is also needed to clarify if prey type influences the ability of TDRs to reliably predict foraging. Results from this study linking verified foraging dives to common dive characteristics potentially opens the door to decades of historical TDR datasets across several otariid species.

## MATERIALS AND METHODS

### Data collection

Data were collected on 11 lactating female Australian fur seals provisioning pups from May-July 2009-2011 at Kanowna Island, Bass Strait, Australia. Kanowna Island is within the Wilsons Promontory Marine National Park and was accessed under permit from Parks Victoria. All work was conducted with approval of the Deakin University Animal Ethics Committee (A16/2008, A14/2011) and under the Department of Sustainability and Environment (Victoria, Australia) Wildlife Research Permits (10005362, 10005484). Seals ranged in mass from 50.5-90.5 kg (mean=73.1±13.9 kg, Table 5). Animals were captured using a hoop net (Fuhrman Diversified Seabrook, Texas, USA) and instrumented with dataloggers while under gas anesthesia (Hoskins and Arnould, 2013). Seals were anesthetized using isoflurane administered with a portable gas vaporizer (Stinger™, Advanced Anaesthesia Specialists, Gladesville, NSW, Australia) and dataloggers were attached to the seal's back along the dorsal midline below the scapula using quick-setting epoxy (Accumix 268, Huntsman Advanced Materials Pty Ltd, Deer Park, Vic, Australia). The fur seals were instrumented with a time-depth recorder (1 Hz, TDR, MK9 or MK10-V, Wildlife Computers, Redmond, WA, USA), an animal-borne video camera (Crittercam, National Geographic Society; Marshall et al., 2007; Marshall, 1998), and a VHF transmitter (Sirtrak Ltd, Havelock North, New Zealand) to assist in relocating the animal for recapture. The Crittercams were programmed to record video when submerged >40 m on a duty cycle of 1 h on and 3 h off. Seals W1873, W1881, W1855, and W1859 were also outfitted with a head-mounted accelerometer (G6A, Cefas Technology Limited, Suffolk, United Kingdom) and a GPS datalogger (FastLoc™1 or FastLoc™2, Sirtrack, NZ) for a concurrent study (Volpov et al., 2015). After full recovery from anesthesia, seals were released into the colony and then recaptured after ≥1 foraging trip using the methods described above. Deployment durations ranged from 3 to 42 days (mean=14±14.6 days), but useable dives for analysis were limited by the duration of concurrent video data (Table 5).

**Table 5.**
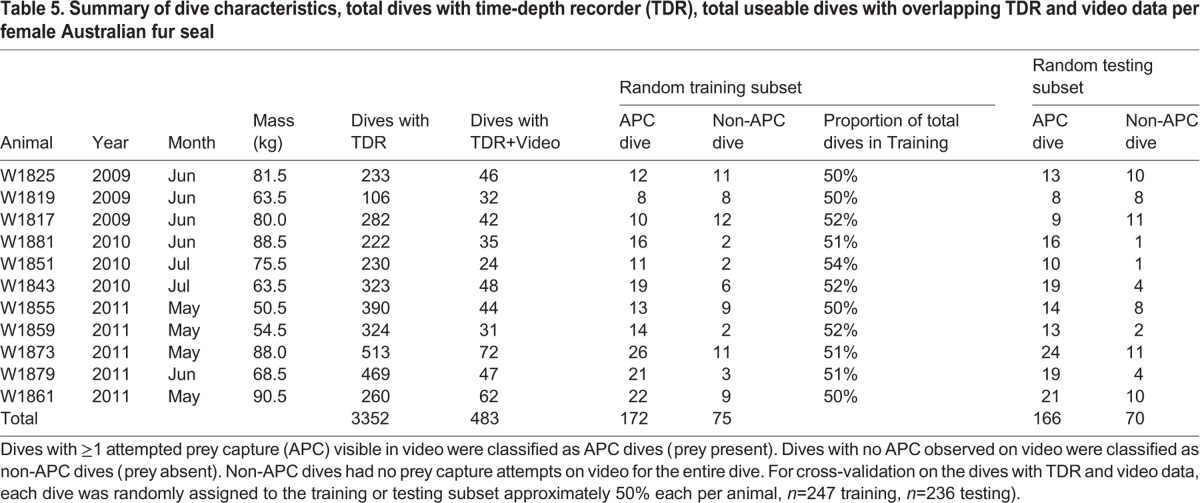
**Summary of dive characteristics, total dives with time-depth recorder (TDR), total useable dives with overlapping TDR and video data per female Australian fur seal**

### Data processing

Video identification of individual attempted APC was performed manually for use in the present study as well as concurrent studies (Volpov et al., 2015). Only dives that had complete descent, bottom, and ascent phases were analyzed. An APC was defined as when a seal attempted to capture one potential prey item visible within the video frame. APCs included both successful (visual confirmation of prey consumption) and unsuccessful capture attempts (prey missed), but similar behaviors that did not include a distinct lunge forward toward prey were excluded. For each individual APC, prey type (fish, stingray, shark, squid, octopus), location of prey consumption (benthically or on ascent), and total prey per dive were classified.

After enumeration at the level of individual APC, data were consolidated at the level of each dive two different ways. First, dives that had at least one APC on video were classified as an APC dive; hence individual APC dives could include multiple APC events. Dives that had no APCs on the video for the entire dive were classified as a non-APC dive. The number of APC dives varied among animals, with 70% of the total dives classified as an APC dive over all animals (Table 5). Second, dives could be classified as either successful or unsuccessful. Successful APC dives were those that had at least one successful APC event (i.e. ingestion of prey), although successful APC dives could include a combination of both successful captures and unsuccessful capture attempts. APC dives that did not have any successful APC were classified as unsuccessful. Unsuccessful dives included both those containing exclusively missed capture attempts within an APC dive and those where no prey were present (i.e. a non-APC dive). The following metrics were calculated: total APC per dive, whether the dive had APC present (i.e. APC or non-APC dive), prey type, if APC was successful or unsuccessful, and location of consumption when applicable (benthic or on ascent). The total APC per dive (both successful and unsuccessful capture attempts) was used as a proxy for foraging intensity. Foraging success refers to analysis of only successful dives or only total successful APC.

The TDR data were zero-offset corrected and then processed with a 40 m minimum dive depth threshold using customized functions in R 3.0.1 (R Core Development Team, 2015) as described in (Volpov et al., 2015). Video and TDR datasets were synchronized to the nearest second using Eon Fusion software (Eonfusion, v.1.2, www.myriax.com) and customized functions in R. The 40 m dive threshold was selected based on the depth of the species' foraging area within Bass Strait being generally 60-90 m and the fact that Australian fur seals are a predominately benthic foraging species (Arnould and Hindell, 2001; Arnould and Kirkwood, 2007). The analysis focused on individual dives rather than dive bouts because video cameras did not sample all consecutive dives for each animal due to the video subsampling schedule and removal of incomplete videos. Previous research also indicates that Australian fur seals might not exhibit distinct dive bouts (Arnould and Hindell, 2001; Arnould and Kirkwood, 2007; Pemberton and Kirkwood, 1994).

Ascent, descent, and bottom portions of the dive were identified using changes in depth slopes. This permitted calculation of the total dive duration, duration of descent, ascent, and bottom phases, ascent rate (m s^−1^), descent rate (m s^−1^), and max dive depth. The proportion of the dive spent in the bottom phase was also calculated. If the proportion of the dive duration in the bottom phase was ≥5% and the dive had no changes in depth during the bottom phase (i.e. wiggles), the dive was defined as ‘U-shaped’ (Arnould and Hindell, 2001). Preliminary analysis on the testing and training subset combined showed that the post-dive surface intervals were heavily skewed right [4.5±30.0 min (mean±s.d.), range=0.8–408.5 min, *n*=510 dives total before threshold applied]. Visualization of the frequency distribution of this dive characteristic showed a significant break in durations at approximately 10 min with 96% of the post-dive surface intervals ≤10 min as also observed in Arnould and Hindell (2001). Consequently, post-dive surface intervals that were >10 min were considered outliers, and likely representative of non-foraging behaviors, and removed from the analyses.

### Statistical analysis

Two-fold cross validation was used to partition the total dives with video for each animal into approximate 50% training and 50% testing subsets (Table 5). Randomly assigning each dive with video to either the training or testing subset accounted for potential temporal, spatial, and prey distribution variation during a foraging trip. Each dive was only used once in the cross-validation process. As the video camera only recorded for 1 h every 4 h when submerged >40 m, video data were considered a random subsample of all dives. These subsampling treatments also mitigated potential autocorrelation during statistical analysis. The training subset was used for selection of which dive characteristics to use as predictor variables on the testing subset. The test subset was used to subsequently validate the model created on the training subset.

Statistical analysis was performed using Generalized Linear Mixed Models (GLMM) and Generalized Additive Mixed Models (GAMM) in R 3.1.2 (lme4 or mgcv packages, Pinheiro and Bates, 2000; R Core Development Team, 2015; Wood, 2006). Extension to GLMMs and GAMMs from Linear Mixed Effect Models (LMEs) was selected in order to model the binomial error distribution and because a non-linear response was expected based on the data. Therefore, GAMMs were initially fitted followed by GLMMs where appropriate. Both GLMM and GAMM utilize individual animal variation relative to the mean of the population while correcting for repeated measurements within and among animals (Zuur et al., 2009). GAMMs are an extension of GLMMs, but GAMMs do not assume a linear relationship and use smoothing on predictive variables (i.e. a GAMM without smoothing is a GLMM; Zuur et al., 2009). Animal ID was treated as a random effect that allowed inferences beyond the sampled population. The most parsimonious model for each research question was fit using a stepwise backwards model selection based on AIC values corrected for smaller sample sizes (AICc). Model validation involved plotting Pearson residuals against fitted values for all covariates in the model and all covariates not used in the model (Zuur et al., 2009).

First, this study determined the probability that a dive had APCs present given a set of potential dive characteristics (i.e. predictor variables) compared with known dive types (APC vs non-APC dive as determined by video) using GLMMs. Second, GLMMs determined the probability that a dive was successful using dive characteristics (i.e. that the dive contained at least one successful APC event). Predictor variable selection for all models was carried out on the training subset (*n*=247 dives). Both of the GLMMs used a binomial error distribution with a logistic link for the response variable of dive type. Predictor variables tested included dive duration, bottom phase duration, post-dive surface interval duration, ascent rate, and descent rate. Given that ascent and descent rates and bottom and dive duration were strongly colinear, these variables were not tested on the same models. There is no direct equivalent of a traditional R^2^ for GLMMs because GLMMs have variance associated with both the random factor (variation between-animals) and residual variance of the fixed factors (within-animal variance). Consequently, model fits were assessed by partitioning variance into the fixed effects (marginal R^2^=R^2^_m_) and random effects (conditional R^2^=R^2^_c_) using the MuMIn package (Wood, 2006) following the methods of Nakagawa and Schielzeth (2013). Additionally, R^2^ values were only reported for GLMMs because this goodness of fit metric was not relevant for GAMMs.

### Accuracy assessment on the testing subset

In order to assess accuracy of using TDR data to reliably predict foraging behavior on dives without video available, the most parsimonious models created on the training subsets were applied to predict the appropriate response variables on the testing subset using only TDR data (i.e. without looking at actual dive type or total APC per dive from video).

For the analysis with dive type as a response variable, each dive was classified as either ‘predicted APC dive’ or ‘predicted non-APC dive’ based on a probability threshold from the GLMM (0.20, 0.40, 0.50, 0.60, 0.65, 0.70, 0.75, 0.90). For example, if a dive had a probability ≥0.50 it was classified as ‘predicted APC dive’ by the TDR data, but if it was <0.50 probability, then it was classified as ‘predicted non-APC dive’. Accuracy was measured as the proportion of dives correctly assigned by the predicted models to either dive type of APC or non-APC (matches=1 point each, incorrect matches=0). The accuracy formula for dive type (APC vs non-APC or successful vs unsuccessful calculated separately) for the GLMM was calculated using Eqn 1 and repeated at each probability threshold.
(1)
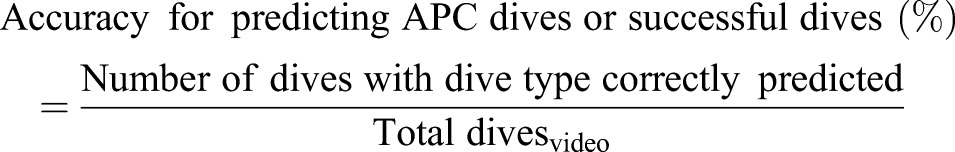

For the analysis with total APC per dive as a response variable, the GAMM created on the training subset was used to predict the total number of APC per dive on the testing subset to yield ‘predicted total APC per dive’. The predicted APC per dive values were rounded down to the lower integer because the video values were integers. For example, predicted APC rates of 1.0 to 1.9 were rounded down to 1 before being compared to the actual total APC on video. Consequently, rounding down yields predictions that are slight underestimates, as opposed to the alternative of rounding up which would yield overestimates. In order to explore if the number of APC per dive influenced accuracy, the predictions were grouped into categories based on each integer value in the dependent variable's range (0-7, eight potential categories). This allowed determination of the proportion of dives correctly predicted (i.e. accuracy) when the model predicted a range of APC per dive.

For each individual dive, the total APC per dive predicted by the TDR data were compared to the total known APC per dive from video separately for each category, using a similar approach as described above. A category was defined according to the predicted total APC per dive on the TDR with 8 distinct categories (noted by *n*) corresponding to 0-7 APC per dive (i.e. all of the dives with 1 APC per dive are noted by category *n*=1). We categorized accuracy by the total number of APC per dive because it is likely that the accuracy would be greater at the mean values compared to the tails due to the distribution of data that the model was built upon (i.e. lower accuracy for less common dives with 5-7 APC per dive). The accuracy in predicting the number of APC per dive was calculated using Eqn 2 for each category (*n*) for the total APC per dive as well as for only the total successful APC per dive.
(2)
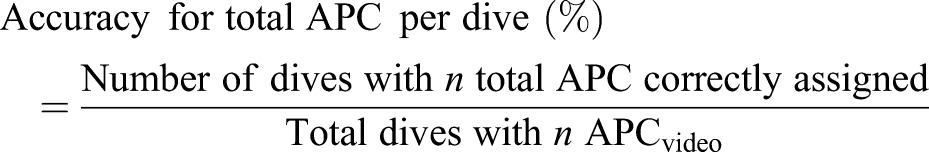

Second, we used GAMMs to investigate the relationship between total APC per dive (successful and unsuccessful) versus each dive characteristic on both APC and non-APC dives. Histograms of the total APC per dive were skewed right and zero-inflated (31% of the 247 APC in the training subset were 0). Consequently, GAMMs were fit with a log link using quasipoisson error distribution to account for the over dispersion in the response variable. This analysis included all dives with a range of 0-7 APC per dive. Third, we used GAMMs to assess the relationship between total number of successful APC per dive and dive characteristics using the same distribution and link function as for total APC per dive. Statistical significance was set at α=0.05. Comparison of mean values for descriptive statistics (i.e. not for GAMM or GLMM modeling) were performed using a mixed effects linear model (LME, nlme package, Pinheiro and Bates, 2000; R Core Development Team, 2015) and likelihood ration test (LRT) on two hierarchically nested models. When the dependent variable is categorical (i.e. APC-dive or non-APC dive), this is analogous to performing repeated measures ANOVA with the important addition of accounting for random effects.
